# Factors Impacting Transgender Patients’ Discomfort with Their Family Physicians: A Respondent-Driven Sampling Survey

**DOI:** 10.1371/journal.pone.0145046

**Published:** 2015-12-17

**Authors:** Greta R. Bauer, Xuchen Zong, Ayden I. Scheim, Rebecca Hammond, Amardeep Thind

**Affiliations:** 1 Department of Epidemiology & Biostatistics, Schulich School of Medicine & Dentistry, The University of Western Ontario, London, Ontario, Canada; 2 Sherbourne Health Centre, Toronto, Canada; 3 Schulich Interfaculty Program in Public Health, The University of Western Ontario, London, Ontario, Canada; 4 Department of Family Medicine, Schulich School of Medicine & Dentistry, The University of Western Ontario, London, Ontario, Canada; David Geffen School of Medicine at UCLA, UNITED STATES

## Abstract

**Background:**

Representing approximately 0.5% of the population, transgender (trans) persons in Canada depend on family physicians for both general and transition-related care. However, physicians receive little to no training on this patient population, and trans patients are often profoundly uncomfortable and may avoid health care. This study examined factors associated with patient discomfort discussing trans health issues with a family physician in Ontario, Canada.

**Methods:**

433 trans people age 16 and over were surveyed using respondent-driven sampling for the Trans PULSE Project; 356 had a family physician. Weighted logistic regression models were fit to produce prevalence risk ratios (PRRs) via average marginal predictions, for transmasculine (n = 184) and transfeminine (n = 172) trans persons.

**Results:**

Among the 83.1% (95% CI = 77.4, 88.9) of trans Ontarians who had a family physician, approximately half reported discomfort discussing trans health issues. 37.2% of transmasculine and 38.1% of transfeminine persons reported at least one trans-specific negative experience. In unadjusted analysis, sociodemographics did not predict discomfort, but those who planned to medically transition sex, but had not begun, were more likely to report discomfort (transmasculine: PRR = 2.62 (95% CI = 1.44, 4.77); transfeminine: PRR = 1.85 (95% CI = 1.08, 3.15)). Adjusted for other factors, greater perceived physician knowledge about trans issues was associated with reduced likelihood of discomfort, and previous trans-specific negative experiences with a family physician with increased discomfort. Transfeminine persons who reported three or more types of negative experiences were 2.26 times as likely, and transmasculine persons 1.61 times as likely, to report discomfort. In adjusted analyses, sociodemographic associations differed by gender, with being previously married or having higher education associated with increased risk of discomfort among transfeminine persons, but decreased risk among transmasculine persons.

**Conclusions:**

Within this transgender population, discomfort in discussing trans health issues with a family physician was common, presenting a barrier to accessing primary care despite having a regular family physician and “universal” health insurance.

## Introduction

Trans (transgender, transsexual or transitioned) persons are those whose gender identity or lived gender varies from their sex assigned at birth [1]. A population-based study from Massachusetts, USA, suggests trans people represent approximately 0.5% of the adult population [2]. Extrapolated to the 2008 population of residents of Ontario, Canada aged 15+, this would represent an estimated 53,500 trans Ontarians.[3] In Canada, management of hormone therapy, and referrals to and coordination with specialists and surgeons, falls within the scope of services provided by family physicians. In Ontario, 67.3% of trans people using hormones accessed them through a family doctor, and only 30.9% through a specialist [4]. Moreover, family physicians play a vital role in prevention and treatment of both general medical conditions and those related specifically to being trans [5]. However, both trans patients and their physicians have identified a lack of trans-relevant clinical training among primary care providers as a barrier to care [1,6,7], given limited coverage within medical education [8].

Under Canada’s provincial public health insurance systems, access to primary care is available free of charge to nearly all residents. The system does not have universal coverage; shortages of family physicians (particularly in rural areas) [9], and socio-cultural access barriers remain [10]. As a result, finding a new family physician can be difficult if one is not satisfied with care. Having a regular family physician and insurance coverage represents potential access to health care, the first step in access. Access must then be realizable (free from further barriers) before it can be realized (actually accessed). Patient-physician communication is considered to be crucial in the process of care, as it impacts both patient satisfaction and outcomes [11]. For trans patients, who may not be identifiable to physicians, communication regarding trans issues is also crucial to realizable access to both transition-related care and trans-competent primary care (the latter also for the 25% of trans people who do not intend to medically transition or are unsure [3]). However, factors that affect patient-physician communication regarding trans-specific health issues have been studied only in very limited ways. Qualitative studies suggest patient comfort discussing trans issues may be impacted by perceived physician knowledge, in the context of limited training [1,6,7].

Trans people have been identified as a medically underserved population that faces stigma within and outside of health care settings [12]. Difficulties in accessing healthcare have been reported in both primary and specialist care settings, including emergency medicine, with some trans persons reporting avoidance of health care due to fears of discrimination [1,13–15]. Frequencies of health care discrimination or avoidance have been shown to vary by age, race, socioeconomic status, gender, and medical transition status.[14–17], with stigma or discrimination contributing to health care avoidance or postponement [14,15] The impacts of this may be compounded by potentially higher need for some health care services; existing research suggests that trans people are at elevated risk for stress related to minority status [18], depression [19,20], suicidality [21], and HIV and other sexually transmitted infections [3,22,23]. Those trans people who need to medically transition require medical care related to hormonal and/or surgical treatments [24]. Thus, facilitating access to trans-competent primary care is critical for the health of this underserved population. For trans people in Ontario, Canada’s most populous province, the current analysis sought to estimate prevalence of, and identify factors associated with, discomfort discussing trans issues with one’s family physician. We hypothesized that the likelihood of discomfort with trans-related physician-patient discussion is shaped by sociodemographic factors, including age, race, marital status and education, as well as trans-specific factors, including prior negative experiences with physicians, history of transphobic experiences generally, perceptions of physician knowledge, and medical transition status.

As research on factors associated with experiences of discrimination and health care avoidance has often been conducted using only transfeminine or only transmasculine participants [15,16], it is unclear to what extent these predictors may vary by gender spectrum. Given prior findings that predictors of other health-related outcomes may vary by gender spectrum [19,20], we hypothesized that predictive factors for discomfort discussing trans issues with a family physician may differ for those on the transfeminine and transmasculine gender spectra.

## Methods

### Survey Methods and Study Sample

As part of the Trans PULSE Project, a cross-sectional survey was conducted in 2009–2010, with data collected from 433 trans participants age 16 or older in Ontario. This remains the only large probability-based data set on trans health and health care in Canada. The survey was multi-mode, completed online or on paper. Participants (including minors age 16 and 17) were asked to indicate informed consent by clicking to begin the survey or by mailing in a completed copy; written signatures were not obtained in order to allow for participant anonymity, if desired. Consent procedures, along with other aspects of the study, were approved by Research Ethics Boards at The University of Western Ontario and Wilfrid Laurier University.

**Fig 1:**
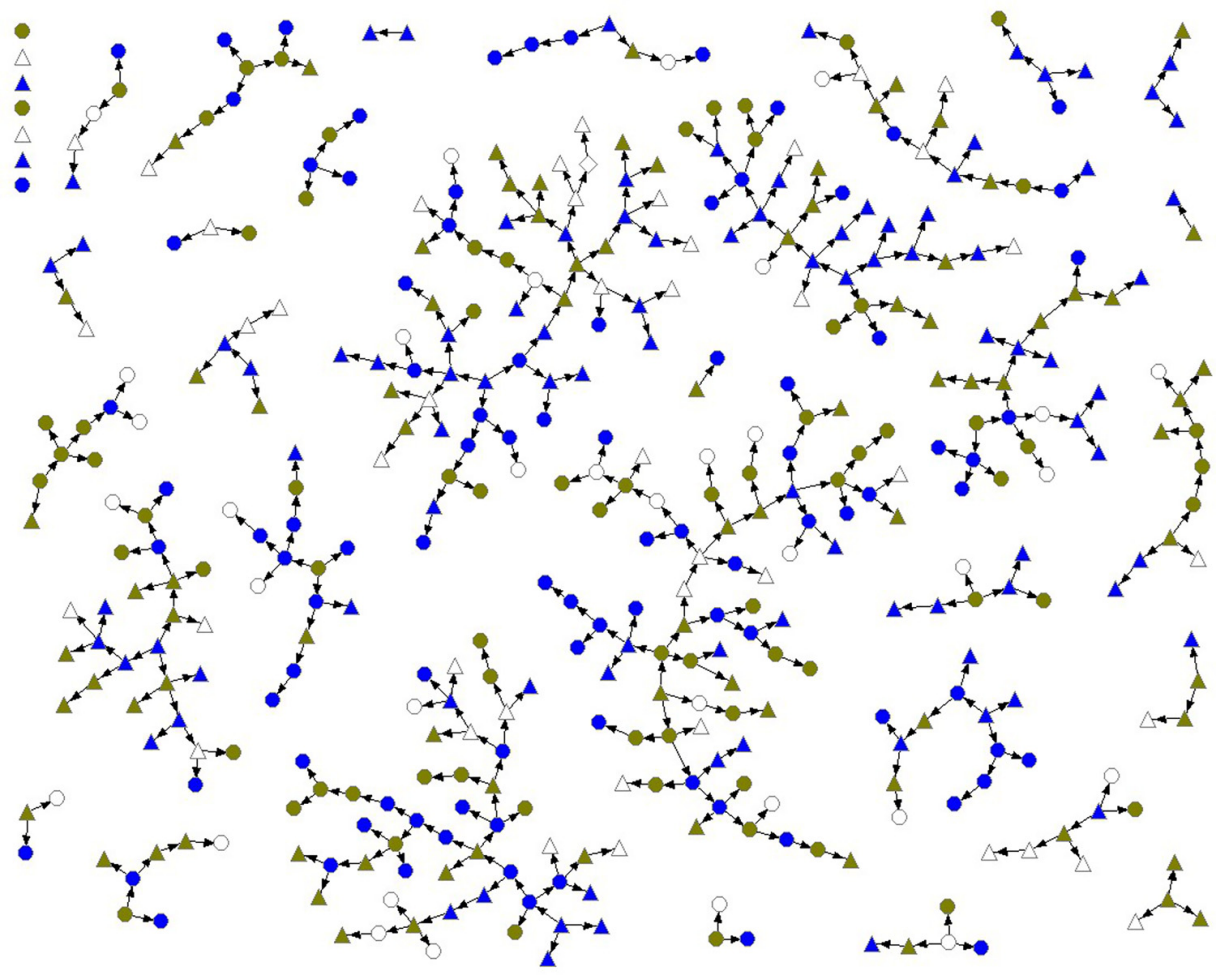
Network diagram of Trans PULSE sample (n = 433), transgender people in Ontario, Canada. Circle = male-to-female or transfeminine spectrum. Triangle = female-to-male or transmasculine spectrum. Gray = has no regular family physician. Olive green = has regular family physician, uncomfortable with discussing trans-related issues. Blue = has regular family physician, comfortable discussing trans-related issues.

Respondent-driven sampling (RDS), a method of tracked chain-referral sampling, was used for recruitment and analysis. RDS is designed to recruit and estimate the characteristics of hard-to-reach or “hidden” populations, those from which a random sample cannot be drawn [25,26]. Recruitment began with 16 seeds, representing a diverse group of original participants, with 22 seeds later added. Each participant was able to recruit up to three eligible peers, who could then each recruit up to three new participants. Recruitment continued until the tenth wave to ensure the attainment of equilibrium (i.e., sample composition stable and independent of the characteristics of seeds). Recruitment patterns were tracked using coupons, and individual network sizes were obtained for use in data analysis. Network characteristics and structure are displayed in Fig 1.

Because some trans people were only connected to trans communities electronically, while others did not have internet connections (or did not have high-speed internet, as was common in Ontario’s rural northern communities), the survey was completed either online (392 participants) or on paper (41 participants), using visually identical survey versions. Online participants received their recruitment coupons online (which could then be printed if needed); paper participants received theirs via paper or e-mail as per their request. Coupons were identically worded and allowed new recruits to either login directly to the online survey or call our toll-free number to request a paper survey. Thus, recruitment chains rarely proceeded through only online or paper chains.

To reduce the probability of duplicate participation though “self-referral”, incentives were limited to a modest primary ($20 gift card for participation) and no secondary (for recruiting others) incentives for 10 months of recruitment; the addition of $5 secondary incentives in the final two months did not alter recruitment detectably. Participants also had the option to donate their $20 honorarium to a trans-related charity. For online participants, IP addresses were recorded, and entries from matching IP addresses checked. If a thread of more than two matching IP addresses had appeared (it did not), the online survey was coded to automatically request the participant to call our toll-free number to receive their incentive, rather than receiving it automatically.

### Measures

All measures were based on self-report. The survey instrument is available online (http://transpulseproject.ca/resources/trans-pulse-survey). Participants reported whether or not they had a regular family physician. For those with a physician, we asked how comfortable they were discussing trans status and trans-specific health care needs with their doctor. Comfort level was dichotomized from a 4-point Likert scale to indicate discomfort (very uncomfortable or uncomfortable) versus comfort (comfortable or very comfortable).

Sociodemographic measures included gender spectrum, age, ethnoracial background, marital status, and education. Participants were classified as transmasculine versus transfeminine spectrum based on sex assigned at birth; participants held a range of current identities, including conventional identities (e.g. “man”, “woman”) and non-binary identities (e.g. “genderqueer”, “Two-spirit”, “bigender”). Ethnicity and race were measured using a check-all-that-apply list and coded as white versus Aboriginal and/or racialized. The term racialized is preferred to “persons of color” in Ontario as it emphasizes race as a social construct.[27] Education was grouped as less than high school, having a high school diploma, some college or university, and having one or more postsecondary degrees.

Trans-specific factors included level of prior negative trans-related (transphobic) experiences, prior trans-specific negative experiences with family physicians, perception of provider knowledge on trans health issues, and medical transition status. Overall levels of transphobic experiences were assessed by an 11-item scale (Cronbach’s α = 0.807), that captured experiences of discrimination (e.g. job loss), microaggressions (e.g. hearing that trans people are not normal), and internalized transphobia (e.g. worrying about growing old alone) [28]. For descriptive frequencies, the level of transphobia experienced was categorized based on the average frequency of experiences reported: low (twice or less), moderate (sometimes), or high (many times). Participants completed a checklist of 9 trans-specific negative experiences with family physicians. A summary variable was created to indicate experiencing none, one to two, or three or more of the nine possible experiences. Perception of provider knowledge on trans health issues was assessed with a 4-point Likert scale. Medical transition status was indicated as completed (could involve a variety of treatments [29]), in process, planning but not begun, or not transitioning. Since few measures have been validated in trans populations, all survey items were pre-tested by the 16 members of the study’s Community Engagement Team for clarity and content validity.

### Data Analysis

All analyses were conducted using SAS version 9.3 [30] and were weighted based on recruitment probability using RDS II methods [31]. Weights were calculated as the inverse of a participant’s network size, and rescaled to sum to the sample total [31]. Variances were adjusted for clustering by shared recruiter to account for non-independence within recruitment chains. Estimates can thus be generalized to Ontario’s networked trans population (i.e. those who know at least one other eligible trans person).

A weighted frequency was estimated for the proportion of Ontario trans people having a regular family physician. The remainder of analyses were conducted for this sub-group (n = 356). Domain analyses were conducted for transmasculine (n = 184) and transfeminine (n = 172) subgroups, to allow for heterogeneity of effects by gender spectrum. Weighted frequencies and associated 95% confidence intervals were estimated for sociodemographic and trans-specific factors, including individual negative experiences with family physicians. Unadjusted and adjusted prevalence risk ratios (PRRs) were estimated to identify factors associated with discomfort discussing trans issues. Logistic regression models were fit using SAS-callable SUDAAN version 11.0 [32]. Average marginal predictions were used to produce PRRs from logistic models [33]. Unadjusted PRRs were estimated for all variables. For each gender spectrum, two multivariable models were fitted; Model 1 included only sociodemographic factors (age, race, marital status, and education), and Model 2 included these factors along with trans-specific factors (transphobia scale score, trans-specific negative experiences with a family physician, perceived physician knowledge about trans issues, and medical transition status).

## Results

Based on our weighted estimate, 83.1% (95% CI = 77.4, 88.9) of trans Ontarians had a family physician. Among those with a family physician, about half of transmasculine (47.7%, 95% CI = 36.6, 58.8) and transfeminine (54.5%, 95% CI = 42.9, 66.1) persons were not comfortable discussing trans issues with their doctor. Frequencies for sociodemographic and trans-specific factors potentially predictive of discomfort are presented in Table 1, for transmasculine and transfeminine Ontarians who reported having a family physician.

**Table 1:** Weighted prevalence estimates among transmasculine and transfeminine spectrum transgender patients with a regular family physician (FP): Ontario, Canada.

	Transmasculine spectrum^a^		Transfeminine spectrum^b^	
	n = 184		n = 172	
	%	95% CI	%	95% CI
**Age**				
16–24	41.0	(30.1, 51.9)	20.9	(10.8, 31.0)
25–44	48.8	(37.5, 59.0)	47.6	(35.5, 59.6)
45+	10.2	(2.7, 17.8)	31.5	(19.4, 43.6)
**Race**				
Racialized or Aboriginal	31.9	(21.2, 42.7)	9.8	(4.9, 14.8)
White	68.1	(57.3, 78.8)	90.2	(85.2, 95.1)
**Marital Status**				
Single (never married)	65.3	(55.1, 75.4)	52.9	(40.4, 65.4)
Married/common-law	24.1	(15.1, 33.1)	16.2	(8.7, 23.6)
Previously married	10.6	(3.4, 17.9)	30.9	(19.2, 42.6)
**Education**				
Less than high school	15.5	(7.5, 23.5)	10.0	(2.2, 17.8)
High school diploma	18.3	(10.3, 26.3)	13.5	(5.0, 22.0)
Some college or university	25.2	(15.2, 35.3)	30.4	(19.1, 41.7)
Postsecondary diploma/degree	41.0	(30.1, 51.9)	46.1	(34.1, 58.1)
**Average transphobia reported** ^c^				
Low	44.5	(32.8, 56.2)	28.0	(17.1, 39.0)
Moderate	47.7	(37.0, 58.3)	57.0	(45.0, 68.9)
High	7.8	(1.7, 13.9)	15.0	(6.3, 23.7)
**Trans-specific negative experiences with a FP**				
None	62.8	(52.9, 72.8)	61.9	(50.4, 73.5)
One or two	24.5	(15.5, 33.6)	23.7	(14.1, 33.3)
Three or more	12.7	(6.1, 19.2)	14.4	(5.9, 22.9)
**Perceived FP knowledge about trans health issues**				
Not at all	31.2	(20.0, 42.4)	40.6	(28.1, 53.0)
Somewhat	38.1	(26.7, 49.4)	31.1	(20.0, 42.1)
Knowledgeable	16.1	(8.5, 23.6)	11.3	(5.2, 17.3)
Very knowledgeable	14.7	(8.8, 20.6)	17.1	(7.5, 26.7)
**Medical transition status**				
Completed transition^d^	31.4	(21.2, 41.5)	24.7	(15.0, 34.3)
In process	17.4	(10.4, 24.4)	35.7	(24.4, 47.0)
Planning but not begun	34.7	(22.9, 46.5)	15.2	(6.9, 23.5)
Not transitioned^e^	16.5	(8.0, 25.1)	24.5	(12.8, 36.2)

^a^. Transmasculine spectrum trans persons include those who were labelled female at birth, and identify as men, or as other masculine or genderqueer/fluid identities

^b^. Transfeminine spectrum trans persons include those who were labelled male at birth, and identify as women, or as other feminine or genderqueer/fluid identities

^c^. Experiences of transphobia—low level: twice or less on average; moderate level: sometimes on average; high level: many times on average.

^d^. Completed transition was based on participant self-report and may involve any combination of hormones or surgery/surgeries.

^e^. Including not planning, not applicable or unsure.

Unadjusted PRRs are presented in the first column in Table 2 for transmasculine persons and Table 3 for transfeminine persons, estimating actual observed differences in prevalence of discomfort between groups. Here, sociodemographic variables were not associated with discomfort discussing trans issues, but some trans-specific variables were. Level of prior transphobia experienced was significantly associated with increased risk of discomfort for transmasculine persons, and decreased risk for transfeminine persons. Medical transition status was significantly associated with discomfort for both gender spectra. Transmasculine persons who were in process of transition, and both transmasculine and transfeminine persons who were planning but had not begun to medically transition, were more likely to report discomfort than those who described themselves as having completed a medical transition. For the transmasculine group only, having had three or more negative trans-specific experiences with a family physician was associated with increased risk of discomfort, and greater perceived physician knowledge about trans issues with decreased risk.

**Table 2:** Prevalence risk ratios for predictors of discomfort discussing trans issues with a family physician: Transmasculine spectrum transgender patients in Ontario, Canada who have a regular family physician (FP) (n = 184).

	Unadjusted associations			Model 1			Model 2		
	Unadjusted PRR^†^	95% CI^†^	P	Adjusted PRR^†^	95% CI^†^	P	Adjusted PRR^†^	95% CI^†^	P
**Sociodemographic factors**									
**Age**			0.4115			0.7093			0.2380
16–24	1.35	(0.85, 2.17)		1.15	(0.69, 1.92)		0.75	(0.52, 1.07)	
25–44	1			1			1		
45+	1.45	(0.65, 3.25)		1.34	(0.66, 2.70)		1.17	(0.77, 1.77)	
**Race**			0.4882			0.7101			0.5996
Racialized or Aboriginal	0.83	(0.47, 1.45)		0.90	(0.52, 1.56)		0.89	(0.56, 1.41)	
White	1			1			1		
**Marital Status**			0.1206			0.2374			**0.0091**
Single (never married)	1			1			1		
Married/common-law	1.15	(0.69, 1.91)		1.21	(0.73, 2.02)		1.08	(0.66, 1.76)	
Previously married	0.35	(0.09, 1.29)		0.51	(0.14, 1.89)		**0.48**	**(0.23, 0.97)**	
**Education**			0.2113			0.4176			0.0631
Less than high school	1.69	(0.94, 3.06)		1.50	(0.84, 2.68)		1.62	(0.89, 2.93)	
High school diploma	1.65	(0.92, 2.96)		1.48	(0.82, 2.68)		**2.01**	**(1.25, 3.22)**	
Some college or university	1.18	(0.60, 2.32)		1.10	(0.58, 2.07)		1.10	(0.70, 1.72)	
Postsecondary diploma/degree	1			1			1		
**Trans-specific factors**									
**Transphobia scale score**			**0.0207**						0.1972
90^th^ versus 10^th^ percentile ^a^	**2.09**	**(1.12, 3.90)**					1.43	(0.84, 2.43)	
**Trans-specific negative experiences with a FP**			**0.0230**						**0.0410**
None	1						1		
One or two	1.33	(0.76, 2.35)					**1.49**	**(1.05, 2.11)**	
Three or more	**1.93**	**(1.27, 2.93)**					1.61	(0.93, 2.79)	
**Perceived FP knowledge about trans health issues**			**0.0041**						**0.0001**
Not at all	1						1		
Somewhat	**0.31**	**(0.16, 0.61)**					**0.37**	**(0.21, 0.65)**	
Knowledgeable	**0.37**	**(0.20, 0.70)**					**0.45**	**(0.28, 0.74)**	
Very knowledgeable	**0.49**	**(0.31, 0.79)**					**0.58**	**(0.37, 0.91)**	
**Medical transition status**			**0.0178**						0.1870
Completed transition^b^	1						1		
In process	**2.19**	**(1.10, 4.36)**					0.99	(0.55, 1.79)	
Planning but not begun	**2.62**	**(1.44, 4.77)**					1.09	(0.67, 1.78)	
Not transitioned^c^	1.97	(0.90, 4.34)					**1.56**	**(1.02, 2.38)**	

^†^ PRR = prevalence risk ratio, here computed from logistic regressions using average marginal predictions; CI = confidence interval

^a^. Experiences of transphobia: 90^th^ percentile = 23; 10^th^ percentile = 5.

^b^. Completed transition was based on participant self-report and may involve any combination of hormones or surgery/surgeries.

^c^. Including not planning, not applicable or unsure.

**Table 3:** Prevalence risk ratios for predictors of discomfort discussing trans issues with a family physician: Transfeminine spectrum transgender patients in Ontario, Canada who have a regular family physician (n = 172).

	Unadjusted associations			Model 1			Model 2		
	Unadjusted PRR^†^	95% CI^†^	P	Adjusted PRR^†^	95% CI^†^	P	Adjusted PRR^†^	95% CI^†^	P
**Sociodemographic factors**									
**Age**			0.9717			0.6091			**0.0113**
16–24	0.93	(0.51, 1.69)		1.26	(0.82, 1.93)		**1.47**	**(1.12, 1.92)**	
25–44	1			1			1		
45+	0.98	(0.58, 1.66)		1.02	(0.68, 1.53)		1.28	(0.91, 1.80)	
**Race**			0.7011			0.8081			0.4264
Racialized or Aboriginal	1.11	(0.67, 1.83)		1.05	(0.71, 1.55)		1.15	(0.83, 1.61)	
White	1			1			1		
**Marital Status**			0.1797			**0.0311**			**0.0032**
Single (never married)	1			1			1		
Married/common-law	0.91	(0.48, 1.74)		1.02	(0.60, 1.74)		0.79	(0.45, 1.37)	
Previously married	1.46	(0.97, 2.21)		**1.67**	**(1.14, 2.45)**		**1.49**	**(1.03, 2.14)**	
**Education**			0.1556			0.1734			**<0.0005**
Less than high school	0.33	(0.08, 1.38)		0.38	(0.06, 2.33)		**0.11**	**(0.03, 0.36)**	
High school diploma	0.62	(0.24, 1.62)		0.69	(0.33, 1.42)		**0.37**	**(0.14, 0.96)**	
Some college or university	1.05	(0.69, 1.61)		1.24	(0.87, 1.76)		0.86	(0.63, 1.18)	
Postsecondary diploma/degree	1			1			1		
**Trans-specific factors**									
**Transphobia scale score**			**0.0174**						**0.0151**
90^th^ versus 10^th^ percentile ^a^	**0.50**	**(0.27, 0.89)**					**0.54**	**(0.32, 0.91)**	
**Trans-specific negative experiences with a FP**			0.4614						**<0.0005**
None	1						1		
One or two	0.80	(0.44, 1.43)					1.38	(0.98, 1.93)	
Three or more	1.24	(0.75, 2.08)					**2.26**	**(1.60, 3.20)**	
**Perceived FP knowledge about trans health issues**			0.1711						**0.0051**
Not at all	1						1		
Somewhat	0.74	(0.45, 1.22)					**0.62**	**(0.43, 0.88)**	
Knowledgeable	0.61	(0.32, 1.15)					**0.55**	**(0.35, 0.86)**	
Very knowledgeable	0.38	(0.12, 1.20)					0.61	(0.35, 1.06)	
**Medical transition status**			**0.0209**						0.5524
Completed transition^b^	1						1		
In process	0.88	(0.45, 1.73)					0.84	(0.58, 1.21)	
Planning but not begun	**1.85**	**(1.08, 3.15)**					1.15	(0.77, 1.74)	
Not transitioned^c^	1.60	(0.89, 2.87)					1.04	(0.73, 1.48)	

^†^ PRR = prevalence risk ratio, here computed from logistic regressions using average marginal predictions; CI = confidence interval

^a^. Experiences of transphobia: 90^th^ percentile = 23; 10^th^ percentile = 5.

^b^. Completed transition was based on participant self-report and may involve any combination of hormones or surgery/surgeries.

^c^. Including not planning, not applicable or unsure.

In multivariable analyses for transmasculine (Table 2) and transfeminine (Table 3) persons, in the models including only sociodemographics (Model 1), being previously married was associated with discomfort discussing trans issues among transfeminine persons, and no other variables were significantly associated. In the models controlling for both sociodemographic and trans-specific factors (Model 2), marital status and education became significantly associated with discomfort for both gender spectra, but in opposite directions. For transmasculine persons, being previously married (versus single and never-married) was associated with a 52% reduction in likelihood of discomfort (RR = 0.48; 95% CI = 0.23, 0.97), whereas for transfeminine persons it was associated with a 49% increase (RR = 1.49; 95% CI = 1.03, 2.14). As compared to postsecondary graduates, transmasculine individuals who had a high school diploma were twice as likely to report discomfort (RR = 2.01, 95% CI = 1.25, 3.22), whereas transfeminine persons were 63% less likely (RR = 0.37, 95% CI = 0.14, 0.96). Being a youth aged 16–24 was also independently associated with greater discomfort among transfeminine persons only.

Trans-specific factors were associated with discomfort discussing trans issues for both gender spectra, independent of other sociodemographic and trans-specific factors controlled for in Model 2. Greater perceived physician knowledge about trans health issues was a strong predictor; having a somewhat to very knowledgeable physician was associated with a relative risk reductions ranging from 38% to 63%, depending on gender spectrum and level of perceived physician knowledge. Within the transmasculine group, those who had not medically transitioned (and had no plans to) were 56% more likely to report discomfort than those who had completed transition (RR = 1.56, 95% CI = 1.02, 2.38). Within the transfeminine group, exposure to higher lifetime levels of transphobia remained strongly associated with reduced discomfort. Previous trans-specific negative experiences with a family physician were strongly associated with discomfort, particularly among those on the transfeminine spectrum; those in this group who had experienced three or more of the specified negative experiences (see Table 4 for specific experiences and their frequencies) were 2.26 times (95% CI = 1.60, 3.20) as likely to report discomfort discussing trans issues with their family physician.

**Table 4:** Weighted prevalence estimates for negative experiences with family physicians among transmasculine and transfeminine spectrum transgender Ontarians who have a regular family physician.

Has a family doctor ever…	Transmasculine spectrum^a^		Transfeminine spectrum^b^	
	N = 184		n = 172	
	%	95% CI^†^	%	95% CI^†^
Refused to see you or ended care because you are trans	7.2	(1.6, 12.9)	5.0	(0.0, 10.3)
Used hurtful or insulting language about trans identity or experience	9.0	(3.5, 14.4)	12.1	(4.1, 20.0)
Refused to discuss or address trans-related health concerns	10.6	(5.3, 15.8)	13.5	(5.5, 21.6)
Told you that you are not really trans	8.2	(2.7, 13.6)	10.9	(2.8, 19.0)
Discouraged you from exploring gender	9.1	(3.4, 14.8)	6.6	(0.7, 12.4)
Told you they don't know enough about trans-related care to provide it	24.5	(15.8, 33.1)	29.1	(18.5, 39.7)
Belittled or ridiculed you for being trans	6.8	(1.2, 12.4)	8.1	(1.9, 14.3)
Thought the gender listed on your ID or forms was a mistake	6.2	(2.3, 10.1)	3.8	(0.0, 9.0)
Refused to examine parts of your body because you are trans	4.5	(1.4, 7.7)	6.2	(0.2, 12.2)
At least one of the above	37.2	(27.2, 47.1)	38.1	(26.5, 49.6)

^†^ CI = Confidence Interval

^a^. Transmasculine spectrum trans persons include those who were labelled female at birth, and identify as men, or as other masculine or genderqueer/fluid identities

^b^. Transfeminine spectrum trans persons include those who were labelled male at birth, and identify as women, or as other feminine or genderqueer/fluid identities

## Discussion

To our knowledge, this is the first analysis examining factors associated with trans patients’ access to family medicine specifically. In Ontario, a family medicine-based health system and “universal” public health insurance without co-payments reduce barriers to physician care. In 2011, an estimated 90.9% of Ontarians had a regular family doctor [34], compared to 83.1% for Ontario’s trans population in our results. Thus, trans people may be somewhat less likely to have a regular doctor, making them more dependent on walk-in clinics.

Having a physician represents potential for health care access, which may or may not be realized depending upon a patient’s willingness and ability to access care, and the quality of that care relative to the patient’s needs. The present study provides a first analysis of factors impacting realizable access to family physicians for trans people. Our estimate that half of trans patients report being uncomfortable discussing their trans status or trans health issues with their regular family physician indicates cause for concern. There is little available empirical evidence with which to compare our results. In a needs assessment of trans people in Virginia (n = 350), conducted using a convenience sample [35], 26% of those with a primary care provider reported being uncomfortable discussing trans-specific health care needs with their provider. Our higher proportion may reflect the broad nature of our RDS sample, in contrast to convenience samples which likely over-represent well-connected trans people who may have better access to trans-friendly health care.

Sociodemographic associations differed markedly across gender spectra, highlighting the importance of conducting analyses either stratified by gender spectrum or tested for interactions. In particular, after controlling for all other sociodemographic and trans-specific characteristics, higher educational attainment and being previously married were associated with increased likelihood of discomfort discussing trans status with physicians among transfeminine persons, but reduced likelihood among transmasculine persons, indicating qualitative effect-measure modification. It is possible that these interesting findings are the result of chance and a peculiarity of our data set, and we would recommend further study of such effect modification. We do not know much regarding how relationship status or dissolution may differentially impact transmasculine versus transfeminine persons or the social support they receive. While it has been shown that transfeminine persons are more likely than transmasculine persons to experience loss of employment and violence [14], which may impact expectations of poor treatment in other settings, job loss and violence were included within our transphobia scale, which was controlled for in this analysis. It would be of interest to explore whether those who experienced positions of greater social privilege prior to transition (e.g. trans women who previously lived as married men and/or well-educated men), and then experienced loss of status, may have less experience and comfort with navigating patient-physician interactions from a socially disadvantaged position.

Of those who reported having a family physician, we estimated that 37.2% of transmasculine and 38.1% of transfeminine persons had prior trans-specific negative experiences with family physicians. While this represents a sizeable proportion of trans patients, frequencies for individual negative experiences were generally lower than those reported within the same data set for comparable experiences in emergency department setting [13]. A total of 31.2% of transmasculine and 40.6% of transfeminine persons perceived their physician to be not at all knowledgeable about trans health issues. Adjusting for all other sociodemographic and trans-specific factors, both prior trans-specific negative experiences and the perception of limited provider knowledge were predictive of discomfort discussing trans issues within both gender spectra. This is consistent with qualitative research in which trans participants link provider education/knowledge with comfort or access [1,6], as well as with quantitative findings that transfeminine persons with experiences of perceived discrimination in healthcare settings were twice as likely to avoid healthcare as those without such experiences [15].

More surprising was the finding that higher levels of transphobia were independently and negatively associated with discomfort among transfeminine persons, controlling for all other factors in the analysis; this may indicate the development of resilience and confidence in response to social stigma, particularly where the experiences were not directly related to prior experience in medical settings (which was controlled). Confounding by social transition status could be a plausible alternative interpretation (those who were “out” as trans may have been more likely to both be comfortable discussing trans issues with others, and to experience transphobia). However, inclusion of a social transition status variable in Model 2 had no substantial impact on results (not shown), demonstrating no support for this possible explanation.

In adjusted analysis, medical transition status was only associated with discomfort for transmasculine persons, among whom discomfort was more likely for those who had not medically transitioned (and had no plans to), as compared to those who had completed medical transition. This may be related to reduced gender identity affirmation experienced by trans people who do not wish to medically transition. We note that in unadjusted analysis, discomfort was more likely among those who were planning to medically transition (for both gender spectrum groups), but had not begun, representing an actual higher prevalence of discomfort in this group. Among this group, discomfort discussing trans issues with one’s physician could reflect anxiety about the potential for the physician to restrict or deny access to transition-related care, and could present an obstacle to beginning a planned medical transition.

### Limitations

Our findings need to be interpreted in the context of the limitations of this study. Since our study included questions regarding experiences with a “regular family physician”, it is unclear to what extent our results would apply to other primary care providers, such as physicians at walk-in clinics or nurse practitioners. As with all studies, it is possible that some findings are chance results, and are due to peculiarities in this particular data set. Our measures also had limitations. We note that our measure of previous negative experiences with a family physician did not distinguish between experiences with past or current physicians, which would have aided in interpretations of our findings. Given the lack of validated measures for trans-specific constructs, many of the measures used in our study were derived by our research team. The list of trans-specific negative experiences did not encompass many experiences that may be common; for example, we asked if a provider had refused to examine parts of a participant’s body because they’re trans, but not whether they had asked to examine body parts not relevant to the issue for which one was seeking care. Also, all items are self-report. We note that patient perceptions of physician knowledge do not necessarily reflect actual physician education. Nevertheless, patient perceptions are relevant even if physicians were indeed well informed, given their demonstrated association with patient discomfort and its potential impact on quality of care.

### Implications

The high proportion of trans patients who reported discomfort discussing trans issues highlights the need to support health care policy-makers and providers in creating trans-inclusive environments and providing integrated and comprehensive services that actively address trans health needs in primary care settings.

The trans-specific negative experiences we have documented may provide some guidance as to areas for action. Trans-specific negative experiences that are hurtful or awkward, such as having a physician think a sex designation on an identity document or record is a mistake, or use insulting language, may impact a patient’s future comfort and likelihood to access care. Physician resources for cultural humility in the care of trans patients are available [36], and recently published practical recommendations for electronic medical records may also help alleviate some of these issues [37–39]. Other negative experiences go well beyond awkwardness and may directly compromise quality of care. That trans patients reported having family physicians refuse to discuss trans issues, refuse to examine parts of their bodies, or end care because they were trans suggest violations of physicians’ duty to care.

The most common negative experience with a family physician reported by trans patients was being told the provider did not know enough about trans-related care to provide it for them. It was not clear to what extent this was an accurate assessment of lack of training in transition-related care, versus a reflection of situations where a physician assumed that trans-specific knowledge was necessary for general care. A tendency for providers to attribute potentially unrelated health conditions to gender or to hormone treatment was documented in this study’s initial qualitative phase [1]. This points to the importance of incorporating trans cultural humility and clinical care needs into existing medical education for family physicians. For example, trans sensitivity education could be incorporated into existing cultural competency/humility modules, while basic hormone treatment information, as well as guidance on when specialist consultation is indicated, could be included in endocrinology-related curricula. Such education is recommended by existing trans care guidelines [36], and supported by the Canadian [40] and American Medical Associations [41].

Overall, our findings show that even in a context of universal basic health insurance, barriers to trans-specific primary care remain. That trans persons were somewhat less likely to have a regular family physician and that half of these reported discomfort discussing trans-specific issues with their physician highlights the distinction between potential access and realizable access within a system where basic transition-related care (i.e. hormone therapy, as well as referrals for any surgical needs) is generally provided in the context of family medicine.
